# Genomic Variants and Multilevel Regulation of *ABCA1*, *ABCG1*, and *SCARB1* Expression in Atherogenesis

**DOI:** 10.3390/jcdd8120170

**Published:** 2021-12-02

**Authors:** Alexandra V. Rozhkova, Veronika G. Dmitrieva, Elena V. Nosova, Alexander D. Dergunov, Svetlana A. Limborska, Liudmila V. Dergunova

**Affiliations:** 1 Department of Molecular Bases of Human Genetics, Institute of Molecular Genetics of National Research Center “Kurchatov Institute”, 123182 Moscow, Russia; avrojk@yandex.ru (A.V.R.); veronuska@mail.ru (V.G.D.); el.nosova94@mail.ru (E.V.N.); limbor@img.ras.ru (S.A.L.); lvd@img.msk.ru (L.V.D.); 2 Laboratory of Structural Fundamentals of Lipoprotein Metabolism, National Medical Research Center for Therapy and Preventive Medicine, 101990 Moscow, Russia

**Keywords:** *ABCA1*, *ABCG1*, atherosclerosis, cholesterol efflux, gene expression, *SCARB1*

## Abstract

Atheroprotective properties of human plasma high-density lipoproteins (HDLs) are determined by their involvement in reverse cholesterol transport (RCT) from the macrophage to the liver. *ABCA1*, *ABCG1*, and SR-BI cholesterol transporters are involved in cholesterol efflux from macrophages to lipid-free ApoA-I and HDL as a first RCT step. Molecular determinants of RCT efficiency that may possess diagnostic and therapeutic meaning remain largely unknown. This review summarizes the progress in studying the genomic variants of *ABCA1*, *ABCG1*, and *SCARB1*, and the regulation of their function at transcriptional and post-transcriptional levels in atherosclerosis. Defects in the structure and function of *ABCA1*, *ABCG1*, and SR-BI are caused by changes in the gene sequence, such as single nucleotide polymorphism or various mutations. In the transcription initiation of transporter genes, in addition to transcription factors, long noncoding RNA (lncRNA), transcription activators, and repressors are also involved. Furthermore, transcription is substantially influenced by the methylation of gene promoter regions. Post-transcriptional regulation involves microRNAs and lncRNAs, including circular RNAs. The potential biomarkers and targets for atheroprotection, based on molecular mechanisms of expression regulation for three transporter genes, are also discussed in this review.

## 1. Introduction

**Figure 1:**
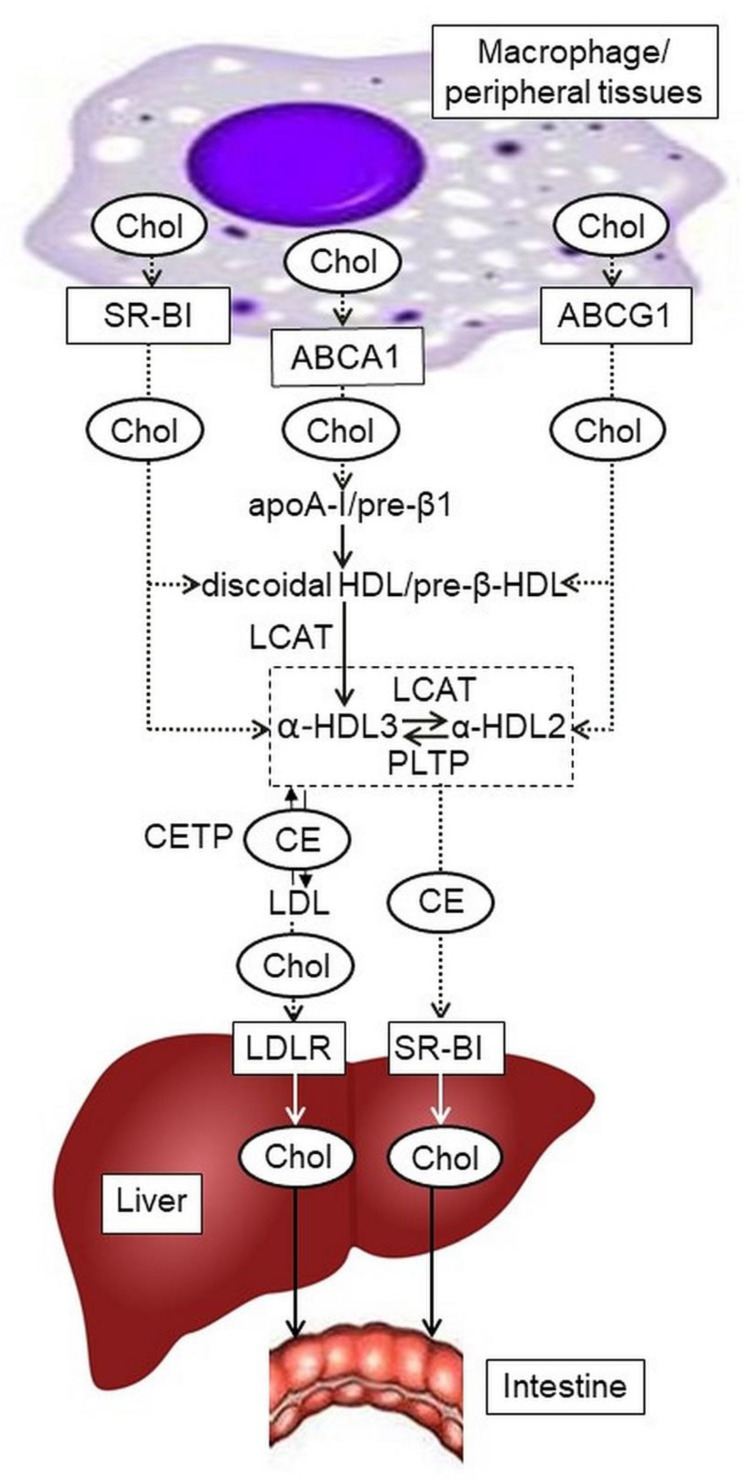
The major steps in the reverse cholesterol transport (RCT) pathway. RCT denotes cholesterol movement from peripheral tissue cells, macrophages in particular, to the liver. The dash arrows correspond to cholesterol transport by ABCA1, ABCG1, and SR-B1 transporters to different cholesterol acceptors and solid arrows correspond to the transitions between different lipoprotein structures. Lipid-free/lipid-poor apoA-I is an exclusive acceptor of cholesterol and phospholipid molecules exported by ABCA1, while both nascent discoidal HDL and mature spherical HDL2 and HDL3 particles accept lipid molecules exported by ABCG1 and SR-B1. The initial complex of apoA-I, with a few molecules of cholesterol and phospholipid with pre-β1-mobility at agarose gel electrophoresis, continues to accept more cholesterol and phospholipid molecules and transforms to a discoidal HDL particle with pre-β-mobility. Discoidal HDL is an efficient substrate for cholesterol esterification catalyzed by lecithin-cholesterol acyl transferase (LCAT) with the appearance of HDL3 and HDL2 as the first and the second products with α-mobilities in sequential reactions of HDL maturation. The backward regeneration of HDL3 from HDL2 particles with the concomitant dissociation of lipid-free apoA-I is catalyzed by phospholipid-transfer protein (PLTP). Cholesteryl ester (CE) molecules in both HDL3 and HDL2 particles are selectively removed by the SR-B1 molecule in the hepatocyte membrane (direct RCT) or exchanged with LDL on triglyceride by cholesteryl ester transfer protein (CETP). CE-enriched LDL particles bind to the LDL-receptor (LDLR) in the hepatocyte membrane and internalize (indirect RCT). Finally, cholesterol in both unmodified and modified to bile acid forms enters the intestine for subsequent excretion with feces.

Despite years of scientific community efforts in treating and preventing cardiovascular disease, atherosclerosis remains the primary cause of the most significant morbidity and mortality worldwide. Atherosclerosis is a chronic inflammation of the subendothelial layer of an artery with the accumulation of lipids and fibrous elements. The nature of cellular and molecular events in atherogenesis has been elucidated and described [1,2,3,4]. An increase in serum cholesterol is believed to be one of the major risk factors for the development of atherosclerosis in humans [3]. Approximately two-thirds of human plasma cholesterol is carried by low-density lipoproteins (LDL), and one-third by high-density lipoproteins (HDL) [5]. Triglyceride-rich very low and intermediate-density lipoproteins are one of the sources of cholesterol-rich low-density lipoproteins; LDL deliver cholesterol to peripheral cells, and cholesterol turnover is normally balanced by cholesteryl ester formation at cholesterol excess with subsequent cholesterol transport by high-density lipoproteins to the liver [6]. Low HDL-C level as a causal factor for coronary heart disease has been challenged as a result of Mendelian randomization studies [7] and a failure of most clinical trials aimed to have therapeutic benefit at raising HDL-C concentrations [8]. Measurements of the total HDL-C may possess a limited value due to the heterogeneous nature of the HDL structure and function, and the quantity or quality [9] of these particles may diversely vary in the low HDL-C level associated cardiometabolic disease [10]. However, certain HDL species may have a significant atheroprotective role by participating in reverse cholesterol transport (RCT) from macrophages to the liver for subsequent elimination. Molecular mechanisms of RCT and the roles of the main participants are described in a large number of reviews [5,11,12,13,14,15,16]. The first stage of RCT is cellular cholesterol efflux. Cholesterol efflux is inversely associated with the risk of atherosclerotic cardiovascular disease (CVD) [17,18,19,20]. In addition, stimulation of efflux has been shown to lead to the regression of atherosclerotic lesions [21]. The accumulated data from clinical, molecular biology, and biochemical studies, have substantially contributed to understanding the role of RCT in atherogenesis and given rise to treatments of atherosclerosis. The efficiency of cholesterol efflux from cells and the cholesterol movement between HDL particles depends on the abundance of cholesterol transporters in the macrophage membrane, extracellular acceptors, and enzyme and receptor activities (Figure 1). ATP-binding cassette (ABC) transporters ABCA1, ABCG1, and scavenger receptor class B1 (SR-BI) play a key role in cholesterol efflux from macrophages and foam cells in atherosclerotic plaques. Notably, the lipid-free/lipid-poor form of the major HDL apolipoprotein (Apo) ApoA-I is the most efficient acceptor of cholesterol effluxed by ABCA1. However, various HDL particles accept cholesterol effluxed by ABCG1 and SR-B1.

While the biochemical events in the RCT pathway have been studied thoroughly, it is still not clear how changes in the functioning of *ABCA1*, *ABCG1*, and *SCARB1* affect atherosclerosis progression. The normal functioning of cholesterol transporters is conducted and controlled at several primary levels, that is, genomic, transcriptional, and post-transcriptional levels (Table 1).

Changes at the genome level, such as nucleotide substitution in the gene sequence or single-nucleotide polymorphism (SNP) and other mutations, can lead to defects in protein structure and function. Gene function is further regulated at the transcriptional level by proteins that ensure the availability of the DNA sequence for transcription, including methyltransferase and deacetylase enzymes, and by factors involved in transcription initiation, such as long noncoding RNA (lncRNAs), transcription repressors, transcription activators, and transcription factors. At the post-transcriptional level of expression regulation, RNA stability plays a key role. RNA stability can be affected by noncoding RNAs, including lncRNA, microRNAs (miRNAs), and circular RNAs (circRNAs).

Here we review the available data on the expression of *ABCA1*, *ABCG1* and *SR-B1* in patients with atherosclerosis, CVD, and animal models of atherosclerosis. We analyze the data for changes in the sequence of transporter genes and disturbances in regulating their function on transcriptional and post-transcriptional levels that lead to atherosclerosis. The emerging role of noncoding RNAs is also discussed. Furthermore, the present review addresses the medical application of the accumulated data and outlines the clinical importance of biomarkers and targets associated with the expression regulation of *ABCA1*, *ABCG1*, and *SR-B1*.

## 2. ABCA1

The membrane-associated protein ATP binding cassette transporter A1 (ABCA1), which mediates the transport of lipid molecules across membranes, is encoded by *ABCA1*. ABCA1 is an integral membrane protein with a size of 240 kDa and contains 2261 amino acids. This protein comprises two transmembrane domains and forms a channel for ATPase-dependent transport of lipids, including cholesterol, phospholipids, and other lipophilic molecules, across the cell membrane [22]. Experimental studies provide evidence that ABCA1 interacts with ApoA-I, including ApoA-I coupled with phospholipids and cholesterol, and is essential for nascent (pre-β1) HDL biosynthesis, thereby promoting cholesterol efflux from cells of peripheral tissues, in particular, macrophages [23]. Thus, as seen in Figure 1, ABCA1 functions at the initial stage of RCT.

### 2.1. Expression Changes in Atherosclerosis

As a result of the fact that RCT impairment underlies the atherosclerotic process, the expression of the ABCA1 transporter should be changed in atherosclerosis. Indeed, numerous studies have shown altered ABCA1 expression during the atherosclerotic process. The messenger RNA (mRNA) of *ABCA1* was significantly increased, but ABCA1 protein, in contrast to mRNA levels, was significantly reduced in the carotid plaques compared with control arteries [24,25]. It can be assumed that this divergence of changes in the levels of mRNA and protein from *ABCA1* in atherosclerosis is associated with post-translational regulation. According to these studies, the level of ABCA1 is also reduced in the plasma of patients with coronary atherosclerosis [26]. Many factors that affect the transport, activity, and expression of ABCA1 have been described [27]. In addition, it was also suggested that such a decrease in ABCA1 content is associated with its degradation by proteinase MMP-9 [28]. The ABCA1 activity is also regulated by the calpain-mediated proteolytic degradation of the ABCA1 protein [29]. In other studies, the level of *ABCA1* mRNA was also decreased in macrophages of patients with atherosclerosis, and the content of ABCA1 was decreased [30,31]. The authors suggested that the level of *ABCA1* mRNA and the level of ABCA1 in macrophages may be essential factors in the development of atherosclerosis. At the same time, these authors showed that the level of *ABCA1* mRNA is reduced in the leukocytes of patients with atherosclerosis. A decrease in the level of *ABCA1* mRNA was also recently found in the peripheral blood mononuclear cells (PBMCs) of patients with coronary artery disease [32].

Thus, *ABCA1* expression changes in tissues modified and damaged by atherosclerosis, such as plaques, macrophages, and mononuclear blood cells of patients with atherosclerosis. A significant increase in *ABCA1* mRNA levels in macrophages and plaques is accompanied by a decrease in the ABCA1 level, which is considered a result of the post-translational regulation of protease degradation and may play a role in the development of atherosclerosis due to RCT impairment. Together, the changes in expression of *ABCA1* in patients with atherosclerosis and atherosclerotic diseases have been well confirmed experimentally.

### 2.2. Studies of Overexpressing and Knockout Mice

Studies of the overexpression and knockout of *ABCA1* in mice cells provide insight into its role in the pathogenesis of atherosclerosis. Such studies were conducted mainly on models of atherosclerosis with the knockout of crucial participants in its pathogenesis—Ldlr, ApoE, and the use of a specific high-cholesterol diet. Macrophage ABCA1 is a major contributor to cholesterol efflux, and RCT in vivo; ^3^H-cholesterol from labeled *Abca1*^−/−^ macrophages injected into *Abca1*^+/+^ mice has returned to serum, liver, bile, and feces by 50% less compared with controls [33]. However, ABCG1 and SR-BI also promote macrophage RCT in vivo [34,35]. Overexpression of human ABCA1 enhanced macrophage cholesterol efflux to ApoA-I; increased plasma cholesterol, cholesteryl esters, free cholesterol, phospholipids, HDL–cholesterol (HDL-C), and ApoA-I and ApoB levels; and led to the accumulation of ApoE-rich HDL_1_ [36]. Endothelial expression of human ABCA1 in mice on a high-fat, high-cholesterol (HFHC) diet increased plasma HDL-C by 40% and reduced diet-induced aortic lesions by 40% [37]. Overexpression of *Abca1* in macrophages of *Ldlr*^−/−^ mice on a Western-type diet also reduced the level of atherosclerosis [38]. By contrast, bone marrow transplantation from *Abca1*^−/−^ mice to *ApoE*^−/−^ or *Ldlr*^−/−^ mice, that is, selective inhibition of ABCA1 in macrophages, led to an increase of atherosclerosis regardless of the HDL level [39,40,41].

These studies in mice indicate that normal *Abca1* functioning can prevent the development and progression of atherosclerosis and are potential therapeutic targets; however, other transporters also efflux to HDL and make a significant contribution to RCT in vivo.

### 2.3. Expression Regulation

#### 2.3.1. Changes at the Genome Level

The *ABCA1* gene encoding ABCA1 protein is located at 9q31 and contains 50 exons. The changes in the *ABCA1* sequence regulate its expression at the genome level (Table 1). Due to the role of ABCA1 in mediating cholesterol efflux from the cells at the initial stage of RCT, the mutations in its gene, which affect the expression of ABCA1 or lead to defects in its protein structure, should disrupt free cholesterol and phospholipid transport across the plasma membrane, the formation of nascent HDL-C particles associated with the development of atherosclerosis and CVD.

To date, numerous mutations in human *ABCA1*, including many SNPs, have been described and lead to various phenotypic manifestations. The best-known is Tangier disease (TD), which was originally described by Fredrickson et al. in 1961 [42]. TD is an autosomal recessive genetic disorder in which both alleles carry mutations leading to the loss of function of ABCA1 [43,44,45,46,47]. The disease is characterized by the changes in serum levels—an almost disappearing HDL, very low ApoA-I, and decreased LDL, the accumulation of CEs in some tissues, and the impaired functioning of different organs. At the same time, TD is developed in some people with compound heterozygosity of mutations in *ABCA1*. For example, carriers of both nonsense mutation R282X and missense mutation Y1532C in *ABCA1* [48], patients with compound heterozygote intronic mutations c.1195-27G > A ac.1510-1G > A causing aberrant splicing of *ABCA1* mRNA [49], and patients with compound heterozygosity for missense variants p.Arg937Val and p.Thr940Met [50] were diagnosed with TD. All these mutations lead to a severe decrease or loss of function of ABCA1, therefore, their carriers should have reduced cholesterol efflux. Indeed, experiments in vitro confirm that cells expressing these mutations elicit significantly less efflux than the wild-type ABCA1 [45,47,48,50]. Among patients with TD, the percentage of cases with premature coronary artery disease (CAD) is increased, but not in all cases [43,46,47]. Patients with TD can carry different mutations and have a decreased LDL level; therefore, it could be supposed that the risk of premature CAD development depends on both factors: the degree of loss of ABCA1 function and LDL/HDL ratio [51]. Carriers of heterozygote mutations, in only one *ABCA1* allele, are classified as having familial HDL deficiency (FHD), characterized by an HDL level below 50% and reduced level of ApoA-I in serum, and less severe forms of the disease. Many studies have found an increased risk of developing CAD in patients with FHD, while CAD is more common in heterozygotes with lower cholesterol efflux values [51,52,53,54]. In some studies, the association of reduced HDL levels with increased CAD risk in patients with FHD was not found, likely due to mild mutations in *ABCA1* in these patients [55,56,57]. Thus, such a high risk of developing CAD is probably connected to the degree of loss of ABCA1 function and premature atherosclerosis, which are found in most patients with FHD [51]. The importance of the LDL/HDL-C ratio as a predictor for CAD in patients with FHD was also confirmed [58].

Some SNPs of *ABCA1* revealed in studies are described. Polymorphisms rs2230806 (R219K), rs4149313 (M8831I), and rs9282541 (R230C), of *ABCA1* are associated with the development and severity of CAD [59,60,61,62]. Similarly, for some SNPs, a less common variant is often associated with decreased CAD risk. Therefore, the K allele of rs2230806 is significantly associated with a decreased risk of CAD, especially in Asian and Iranian populations and people of European ancestry [59,60,62]. A recent meta-analysis also confirmed the effect of R219K in the *ABCA1* on the level of HDL-C and TG, which may result in different risks of CAD [63]. However, most of the mentioned SNPs of *ABCA1* were not detected through the genome-wide association studies (GWAS) as remarkable factors associated with CVD. The effect of SNPs on *ABCA1* expression depends on their location in the DNA sequence. Some SNPs localize in the promoter or coding region and can be expected to affect the expression of *ABCA1* and consequentially the risk of disease development. This may be because this SNP in the *ABCA1* affects the functionality of HDL particles rather than their number. Less common alleles of −565C/T and −191G/C polymorphisms in the promoter of *ABCA1* also predicted a lower risk of coronary heart disease [61,64]. The I883M variant, SNP in the coding region, is associated with higher HDL-C levels together with an increased risk of CAD development [61,64]. As can be seen from most genome studies presented, mutations in *ABCA1* cause the loss of its function to promote the reduction of cholesterol efflux, HDL levels, and increase the risk of atherosclerosis and CVD.

#### 2.3.2. Changes at the Level of Transcription Regulation

The regulation of *ABCA1* expression at the transcriptional level involves events that affect the binding of the transcription factor to the promoter region of this gene and, thus, can affect the transcription initiation (Table 1). At the transcriptional level, the expression of *ABCA1* can be regulated by enzymes, e.g., methyltransferase, deacetylase, other proteins that affect the transcription initiation, and lncRNA, which can interact with different participants of the transcription initiation. This type of regulation leads to an acceleration or deceleration of *ABCA1* transcription, which affects the rate of synthesis of its protein product.

Methylation of cytosine in the CpG islands of the promoter region impedes the interaction of the binding site in the promoter region with transcription factors that downregulates transcription. Experiments in *ApoE ^−/−^* mice have shown that the increased methylation of the promoter region of *ABCA1* decreases its expression and promotes atherosclerosis development [65]. Histone methyltransferase enhancers of zeste homolog 2 (EZH2) and DNA methyltransferase 1 (DNMT1) are consecutively involved in this methylation. Polycomb protein EZH2 mediates DNMT1 expression activation and methyl-CpG-binding protein-2 (MeCP2) recruitment, stimulating the binding of DNMT1 and MeCP2 to *ABCA1* promoter and promoting *ABCA1* gene DNA methylation and atherosclerosis. The increased methylation of the *ABCA1* promoter was also found in patients with early atherosclerosis [66]. These studies are consistent with those showing that the methylation frequency of this site is a factor in CAD development [67,68]. At the same time, the correlation of the DNA methylation level with the blood HDL level may not be observed.

Furthermore, at the stage of transcription initiation, the central role is played by the transcription factors that interact with specific recognition sites on the gene promoter and ensure the activation or repression of transcription. Nuclear receptors LXR (liver X receptors) and RXR (retinoid X receptor) are the key activators of *ABCA1* transcription. Unlike most receptors located on the cell membrane, nuclear receptors are located in the cell nucleus and are simultaneously transcription factors. Nuclear receptors LXR and RXR, acting as a heterodimer, bind to the DR4 element in the *ABCA1* promoter and activate its transcription [69,70,71,72,73]. LXR/RXR is activated by small hydrophobic ligands, such as retinoic acid and hydroxycholesterol, inducing *ABCA1* expression, cholesterol efflux, and promoting RCT. At the same time, unsaturated fatty acids suppress the stimulatory effects of oxysterols and retinoids on the expression of *ABCA1* mRNA, apparently also through the DR4 element [74,75]. Interestingly, LXR/RXR also activates stearoyl-CoA desaturase, which can generate ABCA1-suppressing monounsaturated fatty acids from their saturated precursors. In this case, the activation of LXR/RXR by saturated fatty acids may decrease the *ABCA1* content due to increased desaturation. This mechanism of ABCA1 reduction is likely to occur in cholesterol-loaded macrophages exposed to saturated fatty acids when the activation of LXR/RXR can counteract the enhanced transcription of *ABCA1* [76]. The pattern of association between LXRα, RXRα, and *ABCA1* mRNA expression was found in carotid plaques rather than controls [24,25].

There is evidence that other proteins play a role in activating *ABCA1* transcription by LXR/RXR. Thus, the deacetylase sirtuin 1 (SIRT1) seems to contribute to the transcription activation of *ABCA1* by LXR/RXR. SIRT1 is a transcription activator for LXRα. Oxidized LDL (oxLDL) promotes lipid accumulation and foam cell formation from monocytes by decreasing the level of SIRT1 that decreases the transcription of its target gene *ABCA1* (Table 1) [77]. In addition, endonuclease EEPD1, encoded by *EEPD1*, the LXR target, promotes LXR-stimulated cholesterol efflux by regulating the abundance of ABCA1 at the plasma membrane [78]. Peroxisome proliferator-activated receptor gamma (PPAR-γ), another nuclear receptor, activates *ABCA1* transcription and promotes cholesterol efflux [79]. Moreover, a transcriptional repressor, a protein product of the zinc finger gene 202 (*ZNF202*), binds to the *ABCA1* promoter and inhibits its activity, downregulating cholesterol efflux [80].

Research in the past decade has shown that lncRNA, a subclass of noncoding RNAs with a length greater than 200 nucleotides, is widely expressed and has a critical role in gene regulation [81]. Depending on their specific interactions with DNA, RNA, and proteins, lncRNAs can regulate the expression of genes, including participation in promoter activation during transcription initiation and splicing, and alter the stability and translation of cytoplasmic mRNAs. The mechanisms of lncRNA biogenesis, localization, and functions in transcriptional, post-transcriptional, and other levels of gene regulation are described in detail in another review [82]. At the transcriptional level, lncRNAs can regulate the expression of genes, including participation in promoter activation during transcription initiation. Thus, lncRNAs localized on chromatin can interact with chromatin modifier proteins, affecting their binding and activity at DNA regions of target genes, such as promoters that lead to activation or suppression of their transcription [82]. The involvement of such lncRNA in the pathogenesis of atherosclerosis has also been found. Studies in mice have shown that lncRNA *MeXis* (macrophage-expressed LXR-induced sequence) plays a role in protecting the body from atherosclerosis; it stimulates macrophage cholesterol efflux capacity to ApoA-I and reduces the formation of atherosclerotic lesions in vivo [83]. *MeXis* interacts with transcription coactivator RNA helicase DDX17 and facilitates its action to enhance LXR-mediated *Abca1* expression. Therefore, *MeXis* promotes the activation of *Abca1* expression, cholesterol efflux, and exhibits anti-atherosclerotic properties. Another lncRNA, growth arrest-specific 5 (*GAS5*), localized in the nucleus of macrophages from the cell line THP-1 (a human monocytic leukemia cell line) and increased cellular apoptosis after their treatment with oxLDL [84,85]. *GAS5* can promote lipid accumulation and inhibit cholesterol efflux in THP-1 macrophage-derived foam cells. Studies in *ApoE*^−/−^ mice have shown that *GAS5* encourages the reduction of cholesterol efflux and HDL level in vivo, while levels of TG, TC, and LDL are increased [84]. This is based on the interaction of *GAS5* with EZH2, the catalytic subunit of the PRC2/EED-EZH2 complex, which methylates “Lys-27” (H3K27me) of histone H3, repressing the transcription of *ABCA1* due to a consecutive pattern: EZH2 induces DNMT1 expression and stimulates its binding to *ABCA1* promoter, thereby promoting *ABCA1* gene DNA methylation. *GAS5* interacts with EZH2 and recruits it to the promoter region of *ABCA1*, which inhibits *ABCA1* transcription and decreases the effectiveness of RCT. *GAS5* knockdown is considered to promote RCT and inhibit the accumulation of intracellular lipids, preventing atherosclerosis progression.

Thus, at the transcriptional level, the expression of *ABCA1* is regulated by lncRNA in different directions—the influence of *GAS5* suppresses *ABCA1* transcription and promotes the development of atherosclerosis, the influence of *MeXis*, by contrast, facilitates and increases the transcription of *ABCA1* and prevents the development of atherosclerosis.

#### 2.3.3. Changes at the Level of Post-Transcriptional Regulation of Expression

##### miRNAs

In the post-transcriptional regulation of *ABCA1* expression, noncoding RNAs, including miRNAs, lncRNAs, and circRNAs, play an essential role (Table 1). MiRNAs are short noncoding RNAs (18–25 nucleotides in length) that can bind to the recognition element on the 3′-untranslated region (3′-UTR) of the *ABCA1* mRNA thereby degrading it or inhibiting its translation and, thus, negatively controlling ABCA1 expression. The human genome encodes over 1800 miRNAs [86]. It is believed that miRNAs can regulate over half of human protein-coding genes [87]. The biogenesis and mechanism of miRNA actions are well understood and described in detail [88,89]. To exert their regulatory function, miRNAs assemble with Argonaute (AGO) proteins into miRNA-induced silencing complexes (miRISCs) and mediate the post-transcriptional silencing of complementary mRNA targets [90]. The miRNA binding sites are usually located in the 3′-UTR of mRNA. The binding of miRNA to mRNA occurs due to the complementarity of the bases and leads mainly to the suppression of their expression. For miRNA binding to the target mRNA, a small region of 6–8 nucleotides, the “seed region”, is critical [91]. The degree of complementarity between this miRNA region and the target mRNA largely determines the mechanism of miRNA-mediated gene silencing [90]. Complete complementarity of the sequences degrades mRNA by catalytically active AGO proteins. The partial mismatch involves the additional AGO protein partners to mediate silencing, and GW182 is one of the most important partners. Silencing occurs through a combination of translational repression, deadenylation, decapping, and mRNA degradation [90]. The noncomplete complementarity of microRNA and mRNA targets determines the miRNA-dependent silencing of complementary mRNA. The ability to inhibit expression with incomplete complementarity of miRNA and mRNA sequences may result in a single miRNA suppressing translation of multiple mRNAs [92]. Individual miRNA modulates (mainly reduces) the expression of hundreds of genes, albeit to a small extent (1.5–2 times) [93,94]. In addition to interaction with mRNA and post-transcriptional regulation of gene expression, miRNA can exert post-translational functions. The direct binding of miRNA to proteins that modulate protein function has been observed recently [95,96]. The mechanisms that modulate miRNA activity, stability, and cellular localization through alternative processing and maturation, sequence editing, post-translational modifications of Argonaute proteins, transport from the cytoplasm, and regulation of miRNA-target interactions were reviewed elsewhere [97]. Many miRNAs play a role in the post-transcriptional regulation of *ABCA1* expression. This is due to the length of the 3′-UTR of the *ABCA1* gene, which is more than 3.3 kb, which is much longer than the average length (slightly more than 1 kb) [98]. Due to the length of 3′-UTR, *ABCA1* includes many binding sites for miRNA [98]. Indeed, more than a dozen miRNAs have already been identified, the target of which is *ABCA1* (Table 2).

The greatest number of studies is devoted to the miR-33 functioning. In humans, there are two isoforms, miR-33a and miR-33b; in mice, there is only one isoform of miR-33, homologous to human miR-33a [212]. In mice, human hepatocytes, macrophages, and some other cells, miR-33 suppress *ABCA1* expression by directly binding to sites in 3′-UTR [212,213,214,215]. As a result, the cholesterol efflux from cells to the ApoA-I protein is suppressed. In mice, miR-33 reduces *Abca1* expression in macrophages and liver cells, plasma RCT and HDL levels, as well as cholesterol levels and *Abca1* expression [212,213]. In green monkeys, miR-33a/b suppresses *ABCA1* expression in the liver and plasma levels of HDL [216]. Studies in mice with specific mutations in the miR-33 binding sites of the *Abca1* 3′-UTR, which prevents targeting by miR-33, revealed increased ABCA1 expression in macrophages and liver, as well as enhanced cholesterol efflux and reduced foam cell formation [217]. It is assumed that miR-33 contributes to a decrease in the stability of *ABCA1* mRNA and suppresses the translation of the ABCA1. Moreover, in *Ldlr^−/−^* mice with bone marrow transplantation from these mice with *Abca1* mutation, a decrease in the rate of atherosclerotic plaque formation was observed, similar to that detected for the same mice with bone marrow transplantation from miR-33^−/−^ mice. Thus, miR-33 has a proatherogenic effect primarily associated with ABCA1. Changes in miR-33 expression are shown associated with atherosclerosis development in some conditions. In abdominal aortic aneurysm tissues, mir-33 overexpression was found accompanied by decreased *ABCA1* [215]. In THP-1 macrophages, proinflammatory cytokines increase miR-33a-5P levels, which inhibits cholesterol efflux from cells mediated by ABCA1 [218]. The level of miR-33a is increased and accompanied by a decreased level of *ABCA1* in monocytes of patients with hypertension [174], in the plasma of patients with untreated hyperlipidemia, who have an increased risk of atherosclerosis [219], and in abdominal aortic aneurysm tissues [215]. In PBMCs and plasma of patients with CAD, miR-33a was also overexpressed [130,160,220]. However, in patients with CAD, the level of miR-33a in plaques was reduced compared with levels in adjacent tissues with atherosclerosis [221]. miR-33b has been suggested to post-transcriptionally regulate *ABCA1* expression in atherosclerotic plaques [210]. A significant upregulation of miR-758 and miR-33b was evidenced in plaques from hypercholesterolemic patients when compared to plaques from normocholesterolemic patients. In contrast, miR-33a expression was not different between “normocholesterolemic” and “hypercholesterolemic” plaques [210].

Studies in miR-33^−/−^ mice, including double *ApoE*^−/−^ knockout, have shown that miR-33 deficiency serves to raise HDL-C, increase cholesterol efflux from macrophages via ABCA1, and prevent the progression of atherosclerosis [222,223]. The use of genetically modified humanized mice showed that miR-33b has a similar effect in *ApoE*^−/−^ mice [221]. A comprehensive analysis of the difference between the function of miR-33a and miR-33b was performed using genetically modified mice. miR-33b was dominantly expressed in the liver and induced increased atherosclerotic plaque [224]. Most studies on *Ldlr*^−/−^ mice using the systemic inhibition of miR-33 revealed that this miRNA promotes the development of atherosclerosis [216,225,226,227]. Experiments to identify the role of miR-33 in the development of atherosclerosis found a somewhat controversial effect on the HDL level. An increase in circulating HDL levels and enhanced reverse cholesterol transport to the plasma, liver, and feces was detected only in some studies [216,228]. Ouimet demonstrated that miR-33 promotes the development of atherosclerosis by suppressing the genes of autophagy and polarization in macrophages without involving RCT [226,227]. A study in *Ldlr*^−/−^ mice with miR-33 inhibition found an upregulated HDL with the ability to promote cellular cholesterol efflux instead of the HDL level increase found by others [225]. In another study, the inhibition of miR-33 in hematopoietic cells only (not systemic) led to the suppression of atherosclerosis in *Ldlr*^−/−^ mice [228]. Some mouse studies indicate potentially harmful effects of systemic miR-33 inhibition due to increased obesity, insulin resistance, and blood triglyceride levels, probably due to the increased expression of genes involved in fatty acid synthesis [228]. In general, the results of all these studies suggest that miR-33 contributes to the development of atherosclerosis in mammals by affecting many processes, including the reduction of *ABCA1* expression, which decreases the RCT rate, at least under certain conditions.

The information on other miRNAs that modulate the expression of *ABCA1* is given in Table 2. It should be noted that most miRNAs usually affect different mRNAs, so their effect on atherosclerosis development may not only be due to their influence on *ABCA1*. Most miRNAs suppress the expression of *ABCA1* by direct interaction with the 3′-UTR of its mRNA. This reduces cholesterol efflux from cells both in vitro and in vivo. This, in turn, promotes the development of atherosclerosis as shown in the *ApoE*^−/−^ and *Ldlr*^−/−^ mouse models of this disease for several miRNAs, including miR-10b, miR-19b, miR-20a/b, miR-92a, miR-144, miR-145, miR-148, miR-188-3p, and miR-302a.

It should be noted that most miRNAs usually target mRNAs of different genes, sometimes in the same regulatory cluster. The effect of such miRNAs on ABCA1 expression may be indirect but may still have importance for atherosclerosis development. The miRNAs directly targeting mRNAs other than *ABCA1*, having an influence on ABCA1 expression (indirect target) were also included in Table 2. For example, mir-212, mir-223, and mir-486 directly target SIRT1, transcriptional repressor Sp3, and histone acetyltransferase-1 (HAT1), respectively, which are involved in the regulation of *ABCA1* expression and, thus, affect its level.

##### LncRNAs

LncRNAs Upregulate ABCA1 mRNA through Competitive Interaction with miRNAs

LncRNAs play an essential role not only in the regulation of transcription but also in the post-transcriptional regulation of gene expression. Some lncRNAs can compete with mRNA for binding to miRNA and decrease the effect of miRNA, which suppresses the expression of target genes and, thus, contribute to increasing the expression of these genes, affecting various processes in the human body [229]. These lncRNAs are considered as competing endogenous RNAs (ceRNAs). LncRNAs can affect the expression of the *ABCA1* as a result of both the interaction with other proteins or DNA and competitive interaction with miRNAs targeted *ABCA1*. LncRNA interacts with binding sites for miRNAs to upregulate ABCA1 expression.

Several lncRNAs with ceRNAs properties are involved in the regulation of *ABCA1* expression (Table 1). LncRNA with ceRNA properties include metastasis-associated lung adenocarcinoma transcript 1 (*MALAT1*). This 8.5-kB lncRNA is located at 11q13 and is expressed in atherosclerotic plaques [230]. Simultaneously, the *MALAT1* expression level was significantly decreased in patients with atherosclerosis and oxLDL-stimulated THP-1 macrophages [231]. At the same time, miR-17-5p is highly expressed in the PBMCs of patients with atherosclerosis and suppressing miR-17-5p can alleviate atherosclerosis in *ApoE*^−/−^ mice [104]. Computer analysis and studies in THP-1 macrophages revealed that *MALAT1* has a conserved miR-17-5p binding site, and miR-17-5p may directly target the 3′-UTR of *ABCA1* [231]. *MALAT1* knockdown increases macrophage oxLDL uptake and downregulates the expression levels of *ABCA1*, but also *SR-B1* and *ApoE.* Thus, *MALAT1* can serve as a “sponge” to absorb miR-17-5p, positively regulating *ABCA1* expression and preventing cholesterol accumulation in macrophages.

LncRNAs that activate ABCA1 mRNA through competitive interaction with miRNAs include cholesterol homeostasis regulators of miRNA expression (*CHROME*). The level of lncRNA *CHROME* was elevated in the plasma and atherosclerotic plaques of CAD patients and was upregulated through LXR in response to excess dietary cholesterol in vivo or cellular cholesterol in vitro [232]. *CHROME* binds a number of miRNAs (miR-27b, miR-33a, miR-33b, and miR-128) associated with cholesterol homeostasis and mediates their destabilization or degradation that upregulates *ABCA1* expression. Overexpression of *CHROME-1*, *CHROME-3*, or *CHROME-7* splicing variants reduced the levels of miR-27b, miR-33a, miR-33b, and miR-128, upregulated 3′-UTR of *ABCA1*, and increased mRNA levels of *ABCA1* and cholesterol efflux from macrophages to ApoA-I acceptors.

LncRNA *GAS5* (growth-arrest specific transcript 5) can upregulate the expression of *ABCA1* by competitively binding with miR-33a-5p [233]. Indeed, the specific binding sites between *GAS5* and miR-33a-5p sequences, and between miR-33a-5p and *ABCA1*, have been verified. In addition, lncRNA Maternally expressed gene 3 (*MEG3*) acts as a ceRNA for miR-361-5p, regulating the expression of *ABCA1* [202]. The 3′-UTR of *ABCA1* mRNA contains miR-361 binding sites. Bioinformatics analysis and studies in vascular smooth muscle cells (VSMCs) identified that lncRNA *MEG3* contains one conserved target site of miR-361-5p and miR-361-5p targeted with 3′-UTR of *ABCA1* mRNA. Thus, *MEG3* is ceRNA for miR-361-5p and further upregulates *ABCA1* expression. The expression of *MEG3* was significantly decreased, and miR-361-5p was upregulated in VSMCs after oxLDL treatment. The experimental data suggest that lncRNA *MEG3* regulates miR-361-5p and attenuates proliferation of VSMCs and apoptosis induced by oxLDL, which are involved in the development of atherosclerosis.

LncRNAs Interacting with Proteins or DNA

At the post-transcriptional level, lncRNAs can form lncRNA-protein complexes isolating these proteins and blocking their function. They can also form base pairs with other mRNAs and recruit proteins involved in the degradation of these mRNAs [82]. Currently, several lncRNAs are found to affect the expression of *ABCA1* at the post-transcriptional level as a result of their interaction with other proteins or DNA. In THP-1 macrophages, oxLDL significantly induced the expression lncRNA *DYNLRB2-2*, which upregulates *ABCA1* expression and stimulates cholesterol efflux [234,235]. The exact mechanism of regulation of *ABCA1* expression by *DYNLRB2-2* is not yet determined, but the involvement of an increase in the level of G protein-coupled receptor 119 (GPR119), glucagon-like peptide 1 (GLP-1) [234], and a decrease of toll-like receptor 2 (TLR2), has been shown [235].

Several ncRNAs have been described that are involved in the downregulation of mRNA *ABCA1* and in the progression of atherosclerosis, including *lnc-HC*. The level of *lnc-HC* also increases in rat hepatocytes in response to high cholesterol. *Lnc-HC* forms a complex with the RNA-binding protein hnRNPA2B1; this complex is further bound to the target mRNA *Abca1* and shortens its life cycle [236]. It is assumed that in this way, *lnc-HC* negatively regulates cholesterol metabolism, increasing the risk of metabolic syndrome, which is a risk factor for cardiovascular diseases.

LncRNA “cyclin-dependent kinase inhibitor 2B antisense non-coding RNA” (*CDKN2B-AS1*), also known as “antisense non-coding RNA in the INK4 locus” (*ANRIL*), is characterized by increased expression in atherosclerotic plaques, the promotion of lipid accumulation, and a decrease in RCT rate in foam cells [237]. Overexpression of *CDKN2B-AS1* led to a significant decrease in ABCA1 protein and cholesterol efflux. The exact mechanism of *ABCA1* expression regulation by *CDKN2B-AS1* has not yet been clarified. However, it has been shown that *CDKN2B-AS1* interact with the *CDKN2B* promoter and form complex recruiting methyltransferase EZH2 and transcriptional repressor CCCTC-binding factor, which increases the level of methylation of the *CDKN2B* promoter region and inhibits its transcription.

The level of lncRNA taurine upregulated gene 1 (*TUG1*) is associated with the development of atherosclerosis. The underlying mechanism is not apparent, but overexpression of *TUG1* downregulates the level of mRNA and protein expression from *ABCA1* [238].

Thus, lncRNAs, namely *DYNLRB2-2*, *lnc-HC*, and *CDKN2B-AS1*, affect *ABCA1* expression as a result of their interaction with other proteins or DNA, being involved to some extent in the pathogenesis of atherosclerosis, contributing to the disease development in the case of downregulation of *ABCA1* expression (*lnc-HC* and *CDKN2B-AS1*) and preventing its development in the case of its upregulation (*DYNLRB2-2*).

##### CircRNAs

CircRNA is a new and relatively poorly studied class of lncRNA, found predominantly in mammalian cells [239,240,241,242]. CircRNAs have a covalently closed structure and are often formed in protein-coding genes during backsplicing. CircRNAs do not undergo the action of exonucleases, have increased resistance, and have the ability to act as ceRNA [243]. CircRNAs with binding sites for miRNAs targeted *ABCA1* also possess ceRNA activity and positively regulate *ABCA1* expression. There is evidence that circRNAs also regulate *Abca1* expression. Bioinformatic prediction and RNA pull-down assays determined that *circDENND1B* absorbs miR-17-5p and promotes *Abca1* expression [244]. Overexpression of *circDENND1B* promotes cholesterol efflux reduced by oxLDL and is negatively related to the foam cell formation and progression of atherosclerosis.

Together, circRNA *circDENND1B* and the considered above lncRNA *MALAT1*, *CHROME*, *GAS5*, *MEG3* function as ceRNAs and bind miRNAs that suppress the expression of *ABCA1*, which increases the level of *ABCA1* mRNA and prevents the development of atherosclerosis.

## 3. ABCG1

The membrane-associated protein ATP-binding cassette subfamily G member 1 or ABCG1 is encoded by *ABCG1*. Protein ABCG1 consists of 203 amino acids, and for functioning as a transporter, most likely must form homo- or heterodimers [245,246]. ABCG1 mediates the transport of lipid molecules, including cholesterol and phospholipids such as sphingomyelin, across cellular and intracellular membranes. ABCG1 is highly expressed in macrophages. Unlike ABCA1, which can efflux cholesterol to both ApoA-I and nascent pre-β1 HDL particles, ABCG1 facilitates cellular cholesterol efflux predominantly to HDL particles and promotes RCT [35,247]. It seems that ABCA1 and ABCG1 operate sequentially to mediate lipid efflux from macrophages to ApoA-I and HDL (Figure 1).

### 3.1. Expression Changes in Atherosclerosis

As ABCG1 is involved in RCT, a change in its expression in atherosclerosis can be expected. Indeed, in patients with atherosclerosis, the mRNA level of *ABCG1*, and the content of ABCG1 in blood macrophages, are significantly reduced compared with controls [248]. In addition, the level of mRNA in the monocytes of patients with occlusive vascular lesions was lower than in patients with a smaller degree of stenosis and in the control group. The authors concluded that the mRNA level of *ABCG1* was inversely correlated with the rate of artery occlusion. These findings are consistent with the earlier results on ABCG1 expression in the study of macrophages from patients with type 2 diabetes, which significantly increases the risk of developing atherosclerosis [249]. This study shows a significant decrease in ABCG1 mRNA and protein levels in macrophages, and a correlated decrease in cholesterol efflux. Other researchers have also shown that the expression of ABCG1 is reduced in PBMCs of CAD patients [32]. Together, these studies indicate that decreased expression of ABCG1 in macrophages contributes to the downregulation of cholesterol efflux to HDL particles and RCT impairment that leads to the development of atherosclerosis.

### 3.2. Studies of Overexpressing and Knockout Mice

ABCG1, along with ABCA1, also contributes to RCT in vivo [35]. Cholesterol efflux from macrophages to HDL specifically requires ABCG1 [250]. There is evidence that for cholesterol efflux from macrophages, ABCG1 acts following ABCA1 when ABCA1-mediated lipid efflux transforms ApoA-I into an efficient substrate for ABCG1-dependent cholesterol efflux [251]. *Abcg1*^−/−^ knockout mice showed the accumulation of a large mass of lipids in macrophages and liver without changing the level of blood lipids [250]. Meurs et al. found that the *Abcg1*^−/−^ effect on atherosclerotic development depends on the lesion size; in early atherosclerotic lesions (<167 × 10^3^ μm^2^), *ABCG1* deficiency causes an increase in atherosclerotic lesion development, but at lesion sizes >167 × 10^3^ μm^2^, the role of *ABCG1* in atherogenesis switches from antiatherosclerotic to proatherosclerotic [252]. Bone marrow transplantation from *Abcg1*^−/−^ mice into *Ldlr*^−/−^ mice fed a Western diet led to a significant decrease in lesion area at 11 weeks, which may be explained by registered induction of *Abca1* and increase of ApoE secretion [253]. Enhanced *Abca1* and decreased *Apoa1* expression in *Abcg1*^−/−^ mice have also been registered by RNA-seq [254].

### 3.3. Expression Regulation

#### 3.3.1. Changes at the Genome Level

*ABCG1* is located on the 21q22.3 chromosome region and contains 23 exons. The regulation of *ABCG1* expression at the genome level includes modifications in the gene sequence, changing its expression (Table 1). To date, no genetic disease caused by *ABCG1* mutations has been documented. However, some polymorphisms identified in the *ABCG1* locus can be functional and affect cholesterol efflux, increasing CVD susceptibility.

The association of several polymorphisms of *ABCG1* with the risk of CAD and its severity has been shown in some studies. Ser630Leu, g.−376C > T, and g.−311T > A variants of the *ABCG1* predicted a risk of myocardial infarction [255]. Thus, Ser630Leu, a mutation leading to an amino acid substitution in ABCG1, increases the risk of developing a myocardial infarction by seven times and coronary heart disease by six times. Moreover, levels of *ABCG1* mRNA were decreased in leucocytes of g.−376C > T heterozygotes versus noncarriers due to reduced binding of the *ABCG1* promoter to transcription factor SP1. In contrast, *ABCG1* polymorphism rs57137919 (−367G > A) showed a significantly decreased risk for CAD and myocardial infarction in a Han Chinese population [256]. This polymorphism is accompanied by the downregulation of *ABCG1* expression; ABCG1 protein was significantly lower in macrophages from patients with AA genotype and AG genotype than patients with GG genotype. *ABCG1* −257T > G polymorphism significantly increases the risk of CAD in Japanese male patients, probably due to decreased transcription activity of *ABCG1* in the G allele of −257T > G polymorphism compared with that in the T allele [257].

Thus, some of these *ABCG1* SNPs have a protective role in the development of CAD and MI, due to the contribution of ABCG1 expression to the RCT capacity. This is also supported by the finding that the cholesterol efflux from cells to HDL, mediated by ABCG1, shows an inverse correlation with lipid accumulation in the coronary artery wall of patients with acute coronary insufficiency [258].

#### 3.3.2. Changes at the Level of Transcription Regulation

The regulation of *ABCG1* expression at the transcriptional level involves methyltransferase and deacetylase. The first can modify the nucleotide cytosine in the CpG islands in DNA, and the second, amino acids in histone proteins. These modifications prevent in the case of cytosine methylation or, conversely, facilitate in the case of amino acid deacetylation in histone proteins the interaction of the binding site in the promoter region with a transcription factor that activates *ABCG1* transcription. The binding of the gene promoter region with a transcription factor initiates transcription. LncRNAs also regulate *ABCG1* expression at the transcriptional level. They interact with different participants of the transcription initiation and affect the binding of the transcription factor to the promoter region.

There are studies showing that the methylation of individual loci in the 5′-UTR or promoter region of *ABCG1* is associated with decreased expression of ABCG1 in blood, increased triglyceride levels, carotid intima-media thickness, and an increased risk of CAD [259,260,261,262]. Moreover, *ABCG1* DNA methylation was found to be negatively associated with baseline HDL-C and the change in HDL-C after simvastatin treatment [263]. This association is apparently based on the finding that ABCG1 mediates cholesterol efflux and the efflux of sphingomyelin and phosphatidylcholine, especially because cholesterol efflux has some dependence on sphingomyelin concentrations [264,265]. Together, methylation of the *ABCG1* promoter region downregulates its transcription, and HDL-C deficit may contribute to atherosclerosis.

Several factors are implicated in initiating the transcription of *ABCG1*. As in the case of *ABCA1*, the nuclear receptor LXR/RXR heterodimer transcription factor can activate *ABCG1* transcription. There is evidence that in cultured macrophages, LXR/RXR heterodimers bound to DR4 element in responsive elements LXRE-A and LXRE-B located in *ABCG1* activate their transcription, increasing cholesterol efflux to HDL [266,267]. In addition, *ABCG1* contains putative binding sites for SP1, PPAR-γ, and nuclear factor κB (NF-κB) [79,268]. PPAR-γ activates *ABCA1* and *ABCG1* transcription and promotes cholesterol efflux [79]. The zinc finger gene 202 (ZNF202) transcriptional repressor binds to ABCA1 and ABCG1 promoters and inhibits their activity, which downregulates cholesterol efflux [80].

SIRT1, known as a transcriptional activator, was also shown to play a role in *ABCG1* transactivation by LXR. oxLDL promotes lipid accumulation and foam cell formation from monocytes by decreasing the level of SIRT1, which is a transcription activator for the LXRαα transcription factor, that decreases the transcription of its target gene *ABCG1* [77].

LncRNAs can affect the expression of *ABCG1* when transcription is initiated. In VSMCs and THP-1 cell lines, oxLDL was found to reduce the expression of lncRNA AC096664.3, which inhibits the expression of the transcription factor PPAR-γ, causing a decrease in the level of the protein ABCG1 and increases cholesterol accumulation in the cells, a crucial element of foam cell formation [269]. Overexpression of lncRNA ENST00000602558.1 downregulated *ABCG1* mRNA and protein expression that promotes decreased ABCG1-mediated cholesterol efflux from VSMCs to HDL and increased lipid accumulation in cells [270]. The mechanism of *ABCG1* expression regulation by ENST00000602558.1 is not determined, but ENST00000602558.1 directly binds to p65, which can bind to the promoter region of *ABCG1*, which probably suppresses its expression. The decrease in the transcription level of *ABCG1* under the influence of various lncRNAs is one of the ways to form the foam cells from VSMCs and, thus, contributes to the pathogenesis of atherosclerosis.

#### 3.3.3. Changes at the Level of Post-transcriptional Regulation of Expression

Noncoding RNAs, including miRNAs and lncRNAs, are involved in the post-transcriptional regulation of *ABCG1* expression (Table 1). Table 2 list miRNAs (miR-10b, 23a, 34a, 128, 378) that inhibit the expression of *ABCG1*. Most of them, miR-10b, 23a, 34a, 378, have been shown to contribute to atherosclerosis development. miR-27a and miR-146a-5p suppress *ABCG1* expression indirectly by regulating the expression of other genes [32,140,271,272]. miR-33 directly interacts with 3′-UTR of *Abcg1* and suppresses its expression, while miR-33 does not regulate *ABCG1* expression in humans [222,223,228,273]. Indeed, the miR-33a–responsive element in the human *ABCG1* gene is degenerate compared with the rodents’ sequences and does not confer miR-33a responsiveness [213,273]. However, miR-33a-5P suppresses the expression of *ABCG1* in THP-1 macrophages [218]. Moreover, several reports identified miR-33b as a suppressor of *ABCG1* expression [210,221,274,275]. Thus, miR-33b reduces the expression of ABCG1 and cholesterol efflux while no general opinion seems to exist on miR-33a influence on ABCG1 expression in humans.

There are some reports demonstrating the role of lncRNA in the regulation of ABCG1 gene expression. As mentioned above, for ABCA1, lncRNA *CDKN2B-AS1* also significantly decreases the ABCG1 level in human foam cells, inhibiting the expression of CDKN2B, which can lead to the suppression of RCT and progression of atherosclerosis [237]. LncRNA *TUG1*, which is associated with the development of atherosclerosis, reduces the expression of not only *ABCA1* but also *ABCG1* at the RNA and protein levels, which probably decreases RCT effectiveness and atherosclerosis progression [238]. Thus, at a post-transcriptional level, lncRNAs are proatherogenic and inhibit the expression of *ABCG1*.

## 4. SR-BI

The membrane-associated protein scavenger receptor class B member 1 or SR-BI is encoded by *SCARB1* located at the 12q24.31 chromosome. The SR-BI receptor has various ligands, such as phospholipids, cholesterol esters, and HDL. As presented in Figure 1, the leading role of SR-BI in atherosclerosis is associated with its ability to bind HDL–cholesteryl esters (HDL-CE) and, thus, mediate the uptake of cholesterol esters by the liver. HDL particles bind to SR-BI on the cell surface, and CEs are selectively delivered to the cell. Besides in the liver, SR-BI is also expressed in macrophages and endothelial cells, where it mediates cholesterol efflux, preventing the formation of foam cells and the development of atherosclerosis. In addition, SR-B1 can bind Lp(a), a proatherogenic lipoprotein particle-containing Apo(a) and LDL, probably through the lipid moiety and mediates its intracellular uptake and plasma clearance [276].

### 4.1. Expression Changes in Atherosclerosis

As SR-BI is expressed not only in macrophages and endothelial cells but also in hepatocytes and, thus, are involved in RCT, its expression probably changes during atherosclerosis development. Indeed, there is evidence for the change of SR-BI expression in patients with atherosclerosis. A study of samples obtained during an autopsy after sudden death revealed an increase in the content of *SCARB1* mRNA in the intima of the aorta with atherosclerotic lesions of varying severity compared with the intima of the aorta without atherosclerotic changes [277]. In the monocytes of patients with hyperalphalipoproteinemia compared with those with hypoalphalipoproteinemia, a decrease in the content of *SCARB1* mRNA and a reciprocal correlation of the level of this mRNA with the level of HDL was revealed [278,279]. These changes in the content of *SCARB1* mRNA in atherosclerosis and related conditions specifically indicate the contribution of expression alterations of *SCARB1* to disease development.

### 4.2. Studies of Overexpressing and Knockout Mice

Studies in mice with *Scarb1*^−/−^ knockout on a high cholesterol diet showed massive accumulation of cholesterol-rich HDL in the circulation, reflecting impaired delivery to the liver [280]. *Scarb1^−/−^* knockout studies in *ApoE*^−/−^ and *Ldlr*^−/−^ mice demonstrated that SR-BI expression protects against atherosclerosis [281,282,283,284]. A significant increase in aortic atherosclerotic lesion area was found in such double knockout mice. Overexpression of SR-BI in atherosclerotic *Ldlr*^−/−^ mice reduced atherosclerosis despite markedly reducing HDL-C levels, likely due to increased HDL-C uptake in the liver [285]. It should be noted that mice with *Scarb1*^−/−^ knockout in the liver developed atherosclerosis to a lesser extent than mice with global knockout of this gene, which also indicates the atheroprotective role of this gene in peripheral tissues [286]. Transgenic mice overexpressing human SR-BI in the liver showed increased plasma clearance of Lp(a) cholesteryl ethers, whereas *Scarb1*^−/−^ knockout mice had decreased plasma clearance [276]. Nevertheless, evidence regarding the participation of SR-BI in mediating the cholesterol efflux from macrophages with *Scarb1*^−/−^ knockout is contradictory and varies from zero or minor contribution SR-BI to the cholesterol efflux to substantial [35,287,288]. In addition, studies in vitro and in vivo have shown the role of *Scarb1* in macrophage phagocytosis of apoptotic cells in atherosclerotic plaques [284,289]. Moreover, *Scarb1*^−/−^ macrophages from *Ldlr*^−/−^ mice on the high cholesterol diet had downregulated mRNA levels of IL-1β, IL-6, TNF-α, matrix metalloproteinase 9 (MMP-9), monocyte chemotactic protein 1 (MCP-1), and p65 of nuclear factor NF-κB that suggest the role of *Scarb1* in reducing inflammation [284].

Overall, studies in mice indicate the importance of the normal functioning of *SCARB1* to prevent atherosclerosis development due to both its ability to contribute to the cholesterol efflux, impede the formation of foam cells, and its ability to mediate uptake cholesterol esters to the liver by binding HDL-CE.

### 4.3. Expression Regulation

#### 4.3.1. Changes at the Genome Level

The changes in the *SCARB1* sequence regulate its expression at the genome level (Table 1). For *SCARB1*, two mutations and a number of SNPs were found to be associated with an increased risk of cardiovascular disease. A homozygous variant of the P376L mutation, in which leucine replaces proline 376 in SR-BI, was found in one patient by sequencing the coding regions of lipid-modifying genes in 328 people with extremely high plasma HDL-C levels [290]. The P376L variant caused an almost complete loss of SR-BI functionality. The authors showed that this variant disrupted the post-translational processing of SR-BI and abolished the selective uptake of HDL-CE in hepatocyte-like cells derived from the induced pluripotent stem cells from a homozygous subject. Cholesterol and ApoA-I levels in HDL were significantly increased in the homozygote and heterozygotes compared with controls [290]. In a representative population, heterozygote carriers of the P376L variant had a higher oxidized HDL and an increased risk of CVD than noncarriers [291].

For the missense mutation (P297S) with the loss of function of *SCARB1*, carriers had decreased cholesterol efflux from macrophages, increased HDL-C plasma levels, and decreased uptake of HDL-C by hepatocytes [292]. Thus, studies of *SCARB1* mutation revealed that despite the plasma elevation in HDL-C, carriers exhibit an increased risk of CAD. Some rare mutations of *SCARB1* were found in people with the high HDL-C and high Lp(a) phenotype [293]. These mutations resulted in a partial or complete reduction in cholesteryl ester uptake from HDL3 in vitro, but their impact on the development of atherosclerosis is not clear.

A number of SNPs in *SCARB1*, such as rs4238001, rs10846744, and rs11057830, are associated with HDL levels and the development of CVD [294,295,296,297,298,299,300]. However, some genome-wide association studies revealed no correlation between CAD haplotypes and the HDL level for several cases [298]. SNPs in *SCARB1* associated with HDL, but not with CAD development, have also been described [301].

It can be concluded that mutations of *SCARB1* with the loss of function, impair SR-BI as an HDL-C receptor, contribute to CVD development, including atherosclerosis, mainly by reducing HDL-C absorption by the liver.

#### 4.3.2. Changes at the Level of Transcription Regulation

Currently, only an indirect effect of methylation on *SCARB1* expression has been detected. The remarkable inhibition of *SCARB1* mRNA and SR-BI protein was revealed in atherosclerotic plaque of ApoE^−/−^ mice and in THP-1 macrophage-derived foam cells connected to the expression of DNA methyltransferase (DNMT3b) and decreased level of transcription factor Sp1 [302]. This decreased expression of SR-BI promotes lipid accumulation in foam cells. Interestingly, the decreased expression of *SCARB1* was independent of DNA methyltransferase activity of DNMT3b and connected with the interaction DNMT3b with the *N*-terminal region of SP1, which prevented SP1 binding to *SCARB1* promoter in foam cells.

Many transcription factors are involved in the *SCARB1* transactivation. In human liver cells, besides LXR/RXR heterodimer, farnesoid X receptor 1 (FXR1) together with LXR can bind to their recognition sites at *SCARB1* sequence and transactivate this gene in a synergistic manner [303]. In addition, in liver cells, *SCARB1* can be transactivated by transcription factors PPAR-γ and liver receptor homolog 1 (LRH1) [304].

LncRNA *MALAT1* is found to regulate *SCARB1* transcription. In THP-1 macrophages, oxLDL promotes activation of *MALAT1* transcription by NF-κB. In turn, *MALAT1* binds to b-catenin, the transcription coactivator, and promotes its accumulation on the binding site of the *SCARB1* promoter that activates its transcription probably through TCF4 and PPAR-γ and upregulation of lipid uptake [305,306]. Liu et al. also reported that *MALAT1* knockdown increases macrophage oxLDL uptake and downregulates the expression of *SCARB1* mRNA and SR-BI [231]. Thus, lncRNA *MALAT1* affects the expression of *SCARB1* as a result of interaction with other proteins, contributes to its transcription activation, and prevents atherosclerosis development. Together, the transcription activation of *SCARB1* is regulated by many transcription factors, in particular, LXR/RXR, FXR1, LRH1, PPAR-γ, SP1, and lncRNA *MALAT1.*

#### 4.3.3. Changes at the Level of Post-transcriptional Regulation of Expression

In the post-transcriptional regulation, some miRNAs, which target *SCARB1* and suppress its expression, are associated with cholesterol accumulation underlying atherosclerosis. A detailed description of the miRNAs that regulate *SCARB1* expression is given in Table 2. These are miR-24, -96, -125a, -185, -223, and -455. These miRNAs directly affect the 3′-UTR of *SCARB1* mRNA and inhibit its expression, consequently downregulating HDL-CE uptake by liver cells. Upregulation of these miRNAs in humans could lead to atherosclerosis development. However, the increase in the level of atherosclerosis is shown only for miR-24. miR-223 promotes the accumulation of cholesterol in liver cells, which may increase the risk of developing atherosclerosis. The role of other miRNAs that suppress SR-BI expression in the development of this disease may still be established.

## 5. Medical Application of Data on the Transporter Genes Functioning in CVD

Transporters are essential participants in RCT, and the impairment of their functioning contributes to the development of atherosclerosis and CVD. The experimental data described above for the features of the transporter genes functioning in atherosclerotic RCT disruption can be assumed to identify new targets, which can be used to diagnose and treat atherosclerosis. Such targets can be selected at both transcriptional and post-transcriptional levels of transporter gene expression regulation.

### 5.1. Transcriptional Regulation of Expression

Substances that alter the expression of genes involved in CVD development at the transcriptional level can be considered potential drugs for this disease. Currently, the most intensely studied is apabetalone (RVX-208), a selective inhibitor of proteins containing bromodomains and extraterminal domains (BET proteins), an epigenetic regulator of gene expression, and the driver of atherogenesis [307]. RVX-208 has been shown to inhibit the development of atherosclerosis in ApoE^−/−^ mice [308]. The anti-atherogenic activity of the BET inhibitor, RVX-208, was manifested through a combination of changes in lipid content and anti-inflammatory activity. RVX-208 treatment upregulates ABCA1, ABCG1, and SR-BI–mediated cholesterol efflux and serum levels of ApoA-I and HDL-C in studies in vivo and in vitro [309]. In patients, oral RVX-208 treatment also increased ApoA-I, pre-β-HDL, and HDL functionality. Apabetalone is also being considered as a candidate for CVD therapy that effectively suppresses inflammation by inhibiting, in particular, IL-8 and TLR2, as well as proteins involved in plaque stability, such as the transcription factor IRF1 in patients with CVD and several other genes involved in atherogenesis [310]. In some clinical trials, apabetalone treatment led to plaque attenuation correlated with HDL and VLDL plasma levels and fewer heart failure hospitalizations in patients with recent acute coronary syndrome ACS [307,311]. However, there are clinical studies in which the use of apabetalone did not significantly reduce the risk of cardiovascular disorders in patients with type 2 diabetes after acute coronary syndrome [312] and the progression of atherosclerosis in patients with CAD [313].

Therapeutic agents acting at the level of gene transcription are of great interest. As the methylation of the promoter region of *ABCA1* contributes to atherosclerosis development [65,66], substances that inhibit methylation can prevent the downregulation of this gene and promote cholesterol efflux. For example, *N*-phthaloyl-l-tryptophan 1 (RG108), a DNMT1 inhibitor, and its maleimide derivatives can be considered as potential agents for the treatment of atherosclerosis and CAD [314,315]. Procainamide, which is used for the treatment of ventricular tachycardia, is also found to be a specific inhibitor of DNMT1 [316,317]. Nanaomycin A, the specific inhibitor of DNMT3b, upregulates the mRNA and protein expression of *SCARB1* in foam cells [302]. Additional DNMT3B inhibitors may also be tested to affect *SCARB1* expression [318].

Taking into account that the downregulation of SIRT1 decreases the transcription of *ABCA1* [77], the activation of SIRT1 may be considered as a potential therapy for atherosclerosis and CVD. Some dietary supplements, including resveratrol, quercetin, and curcumin, upregulate SIRT1. Quercetin enhances oxLDL-impaired SIRT1 expression [319]. Moreover, in a clinical trial of subjects with hypertension, quercetin decreases the plasma level of proinflammatory cytokines IL-1b and E-selectin [320]. Resveratrol has been shown to protect against CVD and reduce the atherosclerotic area in ApoE^−/−^ and LDLR^−/−^ mice [321,322]. Resveratrol suppresses lipid accumulation and foam cell formation from THP-1 macrophages [323]. However, there is evidence that resveratrol is not a direct activator of SIRT1 [324]. Nevertheless, in clinical trials, resveratrol increases serum concentrations of SIRT1 [325], decreases the plasma level of chemokines in healthy subjects [326], and improves left ventricular systolic and diastolic functions in patients with stable CHD [327]. Curcumin has been reported to enhance cholesterol efflux by upregulating ABCA1 expression through activating AMP-activated protein kinase, its downstream target SIRT1, and transcription factor LXRa in THP-1 macrophage-derived foam cells [328]. It should be noted that the protective effect of these dietary supplements on atherosclerosis development is also connected to the inhibition of inflammation, including as a result of SIRT1 activation [329,330,331].

### 5.2. Post-transcriptional Regulation of Expression

#### 5.2.1. MiRNAs

The effect of miRNA on the expression of genes involved in the pathogenesis of atherosclerosis and CVD, including transporters, is well characterized. The inhibition of miRNAs targeting genes encoding the lipid transporters can be considered to treat these diseases. There is a comprehensive overview of the medical application of miRNAs for different therapeutic targets in cardiovascular disease [332].

The beneficial effects of inhibition of a variety of miRNAs have been shown in animal models of CVD. For example, miRNAs miR-19b, miR-34a, and miR-33, as already mentioned, suppress the expression of *ABCA1* and are considered potential targets for CVD therapy in humans [117,147,222,225,333]. For several of these miRNAs, therapeutic molecules MGN-2677, MGN-5804, and MRG-110 inhibiting miR-143/145, -378, and -92a, respectively, are already being investigated [334]. The first in-human study of a locked nucleic acid-based antisense oligonucleotide MRG-110 inhibiting miR-92a was promising: a single intravenous injection dose and time-dependently reduced miR-92a levels [335]. One study has suggested the delivery of miRNAs into macrophages that promote cholesterol efflux from foam cells to the liver for the treatment of CVD [336].

The combined antagonism of miR-148 and miR-128-1 is another promising therapeutic approach to the treatment of dyslipidemia [337]. The inhibition of miR-148a increases liver LDLR expression and decreases plasma low-density lipoprotein-cholesterol levels in mice. MiR-128-1 regulates ABCA1 expression in macrophages and improves cholesterol efflux from them. As miR-320b reduces HDL and ApoA-I mediated cholesterol efflux from macrophages, the inhibitory effect may also be a promising therapeutic target for the treatment of atherosclerosis [338].

Due to the fact that the level of many miRNAs changes in CVD, they can be used to diagnose these diseases. Thus, the expression level of miR-223, which regulates the expression of *ABCA1* and *SCARB1*, can serve as a marker for the diagnosis of CVD [198,339] and for the prognosis of the disease course [193,197]. The European patent EP2925884B1 describes using a biomarker panel of four miRNAs (miR-16, miR-27a, miR-101, and miR-150) to predict a patient’s condition after acute myocardial infarction [340]. Patients with acute myocardial infarction and high miR-16 and mi-R27a, and low levels of miR-150 and miR-101 are more likely to have worse left ventricular contractility than those with normal levels.

The use of new integrated approaches for the treatment of dyslipidemia and CVD is also considered. For some of them, the effect on the expression of transporters and cholesterol efflux is confirmed. *Lactobacillus acidophilus* species are well-known probiotics with beneficial cholesterol-regulating activity. *L. acidophilus* K301 increases the expression of genes, such as *ABCA1* and *ABCG1*, under the control of LXR, resulting in an increase in ApoA-I-dependent cholesterol efflux, suggesting the therapeutic potential of *L. acidophilus* K301 as an anti-atherosclerotic agent [341]. Sonodynamic therapy (SDT) is a novel approach that involves a combination of low-intensity ultrasound and specialized chemical agents known as sonosensitizers. SDT with 5-aminolevulinic acid as a sonosensitizer (ALA-SDT) activates the PPAR-γ-LXRa/ABCA1 and ABCG1 pathway, increasing cholesterol efflux, induces an anti-inflammatory response, and ultimately reduces the signs of atherosclerosis [342].

#### 5.2.2. LncRNAs

Noncoding RNAs are regulators of lipid metabolism, affecting the expression of genes involved in the capture, esterification, and cholesterol efflux, and are also able to regulate inflammatory processes; therefore, they can be considered as promising therapeutic targets for the treatment of atherosclerosis and CAD [343]. Based on the results of experiments aimed at increasing or decreasing the level of antiatherogenic or proatherogenic RNAs, this approach has been suggested for CVD therapy.

Exposure to lncRNAs with antiatherogenic properties, such as upregulation of transporter gene expression and activation of macrophage cholesterol efflux, is a potential therapeutic targeting strategy for atherosclerosis treatment. Among lncRNAs provided in the review, these criteria are met by *MeXis*, *MALAT1*, *CHROME*, and *MEG3*.

Considering lncRNA *GAS5* as a target for the treatment of atherosclerosis in humans, it can be seen that *GAS5* has proatherogenic properties, promoting methylation of the *ABCA1* promoter region [84]. Indeed, *GAS5* suppression stimulates RCT, suppresses intracellular lipid accumulation, and, as a result, decreases the progression of atherosclerosis. *GAS5* also displays antiatherogenic properties interacting with miRNA; *GAS5* can upregulate the expression of *ABCA1* by competitively binding with miR-33a-5p [233]. It should be noted that polymorphism in the *GAS5* promoter region, rs145204276 DEL/DEL, which upregulates *GAS5* transcription activity, was shown to decrease atherosclerosis risk in a Chinese population [344].

Some lncRNAs highlighted in the review affect the expression of transporters at the post-transcriptional level and can be used to predict the development of atherosclerosis and CAD. As the level of some ncRNAs correlates with the outcome of CVD, in particular acute myocardial infarction, the data for the ncRNA expression can also be used to diagnose the disease and predict its course [345,346]. For example, *CDKN2B-AS1* (or *ANRIL*) and *MALAT1* can be used to predict the development of left ventricular dysfunction as *ANRIL* was expressed at lower levels and *MALAT1* expression was higher in patients with myocardial infarction than in healthy volunteers [345].

#### 5.2.3. CircRNAs

For a number of circRNAs, a change in expression was shown in various CVDs. In this regard, some circRNAs can be considered as potential biomarkers of individual CVDs or used to predict the disease course. Thus, the expression level of hsa_circ_0124644 (*ROBO2* gene, roundabout guidance receptor 2) is significantly increased in the peripheral blood of CAD patients and can be used as a diagnostic biomarker of CAD [347].

In addition to its linear form, lncRNA *ANRIL* also has a circular isoform (*circANRIL*) associated with the atheroprotective 9p21 genotype [348]. *CircANRIL* functions were independent of CDKN2B and miRNA absorption and capable of preventing pre-rRNA maturation in macrophages by binding to pescadillo homologue 1, activating p53, and thereby enhancing apoptosis and inhibiting proliferation. Carriers of the CAD-protective haplotype at 9p21 showed significantly increased expression of *circANRIL* in PBMCs [349]. Thus, *circANRIL* appears to be used as a predictor of the positive outcome of CVD. CircRNA named myocardial infarction-associated circular RNA (*MICRA*, gene ZNF609 (zinc finger protein 609)), whose function is currently unknown, but probably connected to miR-150, is associated with the outcome after myocardial infarction; patients with low levels of *MICRA* in the blood were at high risk of left ventricle dysfunction after myocardial infarction [350]. It is assumed that *MICRA* level can be used to predict the development risk of left ventricle dysfunction after myocardial infarction.

Some patents describe circRNAs whose levels correlate with CVD. For example, the inventors of the patent EP3054017A1 revealed that in the level of circRNAs detected by them, U1 (DENN domain containing 4C gene, *DENND4C*), U2 (PDS5 cohesin associated factor A gene, *PDS5A*), and U4 (zinc finger protein 292 gene, *ZNF292*) are increased in the plasma of patients with CAD, and the levels of U1 and U4 in the plasma of patients with acute myocardial infarction. CircRNA data are proposed to be used as biomarkers for the diagnosis of CVD data.

## 6. Conclusions

Changing the function of cholesterol transporter genes has great potential for developing new approaches to anti-atherosclerotic drug therapy. A decrease in the expression of these genes contributes to inhibiting RCT and the development of atherosclerosis, CAD, and CVD, while an increase in their expression prevents the development of these diseases. In that way, the normal functioning of transporter genes, and hence RCT, plays an essential role in preventing the development of atherosclerosis.

Studies of the regulation of *ABCA1*, *ABCG1*, and *SCARB1* expression at transcriptional and post-transcriptional levels have revealed new proteins and ncRNAs involved in their function. Many factors, particularly methyltransferases and miRNAs, suppress the expression of transporter genes and contribute to atherogenesis in humans. For other factors, particularly those involved in the post-transcriptional regulation of the expression of transporter genes, some lncRNAs and circRNAs, anti-atherogenic effects have been shown.

This accumulated knowledge regarding the peculiar problems of transporter gene expression regulation in atherosclerosis and various CVDs can be applied in medicine to diagnose and treat these diseases. The influence on the enzymes responsible for epigenetic modifications at the level of transcription regulation of the lipid transporters is studied as a therapeutic approach to treating atherosclerosis and CVD. Methyltransferase inhibitors have shown promising results in activating the expression of lipid transporters. The most encouraging is the activation of SIRT1 deacetylase by dietary supplements, which also reduces inflammation. As miRNAs targeting *ABCA1*, *ABCG1*, and *SCARB1* encoding the lipid transporters have a suppressing effect on their expression, therapeutic molecules that inhibit miRNAs are being investigated as a potential therapy for these diseases. However, a single miRNA can suppress the translation of many mRNAs, thus regulating a wide list of genes, and the overall effect may be difficult to predict. Therapy with agents based on noncoding RNAs with activating effects on the expression of lipid transporters, particularly some lncRNAs and circRNAs, may be considered as a potential therapeutic strategy for the treatment of atherosclerosis. At the same time, these noncoding RNAs, including circRNAs and miRNAs, are studied as diagnostic biomarkers for atherosclerosis and related CVD and CAD. Thus, most reviewed agents regulating the expression of the lipid transporters, on the one hand, are involved in the pathogenesis of atherosclerosis and related CVD, and on the other hand, are promising targets for the treatment of these diseases.

## Figures and Tables

**Table 1:** Expression regulation levels of three cholesterol transporter genes in different cell compartments known to influence their function.

Expression Regulation Levels (Cellular Compartments)	Participants	*ABCA1*	*ABCG1*	*SCARB1*
Genome (nucleus)				
	SNPs/mutations	●	●	●
Transcription (nucleus)				
	methylation of the promoter region	●	●	●
	transcription activators	●	●	●
	transcription repressors	●	●	
	transcription factors	●	●	●
	lncRNAs	●	●	●
Post-transcriptional regulation (cytoplasm)	lncRNAs interacting with miRNA	●		
	lncRNAs interacting with proteins or DNA	●	●	
	miRNAs	●	●	●
	circRNA	●		

●, known regulation level.

**Table 2:** MiRNAs regulate the expression of *ABCA1*, *ABCG1*, and *SCARB1* genes.

miRNA	Target	Expression Change in Cardiovascular Diseases (CVD) and Knockout and Model Mice	In Vitro Effect on Lipid Level and Reverse Cholesterol Transport (RCT)	In Vivo Effect on Lipid Level, RCT and Atherosclerosis
miR-9	*ABCA1*	Plasma level of hsa-miR-9-3p decreased in patients with unstable angina (UA) [99].	MiR-9-5p directly bound to the 3′-UTR of *ABCA1* and reduced its mRNA and protein levels in macrophages [100].	
miR-10b	*ABCA1/ABCG1*	MiR-10b level increased in atherosclerotic plaques in humans [101].	MiR-10b directly bound to the 3′-UTR of *ABCA1/ABCG1* and suppressed their expression and cholesterol efflux from mouse peritoneal macrophages (MPMs) and human THP-1 monocytes [102].	In *ApoE^−/−^* mice, miR-10b suppressed the expression of *ABCA1*/*ABCG1* and RCT from macrophages to feces, thus contributing to the development of atherosclerosis, the growth of plaques and their instability in the late stages [102,103].
miR-17	*ABCA1*	An increase in the level of miR-17-5p has been found in leukocytes of patients with atherosclerosis [104], in plasma of patients with UA [105], acute myocardial infarction (AMI) [106], CAD [107,108]. The serum level of miR-17-5p was also associated with the development of ischemic heart disease (IHD) [109] and the severity of CAD [110]. miR-17-3p levels also increased in atherosclerotic plaques in humans [101]. However, a decrease in the circulating miR-17-5p level has been found in patients with CAD [111] and CHD [112].	MiR-17-5p directly bound to the 3′-UTR of *ABCA1* and suppressed its expression in mouse macrophage RAW264.7 [104].	The level of miR-17-5p increased in the macrophages of *ApoE^−/−^* mice on a high-cholesterol diet [104].
miR-19b	*ABCA1*	MiR-19b levels elevated in human atherosclerotic plaques and rat aortic tissues of the abdominal aortic aneurysm (AAA) model [113,114], in plasma of patients with AMI [115] and in plasma endothelial microparticles (EMPs) of patients with UA [116].	MiR-19b directly suppressed *ABCA1* expression and cholesterol efflux from MPMs and macrophages derived from human THP-1 monocytes [117].	In *ApoE^−/−^* mice, miR-19b suppressed the expression of *ABCA1*, RCT and the level of HDL in plasma, thus increasing the size of aortic plaques and contributing to the development of atherosclerosis [117,118].
miR-20a/b	*ABCA1*	Changes in miR-20a expression in atherosclerosis-associated diseases are multidirectional. Thus, the level of miR-20a increased in human aorta with AAA [119] and in plasma of patients with UA as well [99,105]. In contrast, the level of miR-20a decreased in blood cells of patients with AMI [120] and in plasma of patients with CAD [111]. MiR-20b was also low in blood cells of patients with the peripheral arterial disease (PAD) [121]. Expression of miR-20a/b decreased in the liver of *ApoE^−/−^* mice on a high fat diet [98].	MiR-20a/b bound to the 3′-UTR of *ABCA1* and suppressed its expression and cholesterol efflux from THP-1- and RAW 264.7-derived foam cells [98].	In *ApoE^−/−^* mice, miR-20a/b reduced *ABCA1* expression in the liver, RCT efficiency and HDL synthesis, thus contributing to the development of atherosclerosis [98].
miR-23a	*ABCA1ABCG1*	Increased values for miR-23a were associated with atherosclerosis-related diseases, i.e., an increased miR-23a level has been detected in the plasma of patients with acute ischemic stroke (AIS) with vulnerable carotid plaques [122], in plasma of patients with UA [99] and in plasma and PBMCs of patients with CAD [123,124,125,126]. miR-23a levels are correlated with plaque development [122], stenosis degree [123] and poor clinical outcomes in CAD [124]. OxLDL upregulated miR-23a expression in macrophages [122]. However, miR-23a level in plasma decreased within 24 h of stroke onset in humans [127].	MiR-23a suppressed the activity of 3′-UTR of *ABCA1* and *ABCG1*, reduced their expression and cholesterol efflux, that led to foam cell formation [122].	In *ApoE^−/−^* mice, miR-23a suppressed *ABCA1* and *ABCG1* expression, promoted atherosclerosis and increased plaque vulnerability [122].
miR-24	*SCARB1*	The data are contradictory. Fatty acids increased the expression of miR-24 in HepG2 cells. The miR-24 levels significantly increased in the liver of obese mice [128], in the plasma of patients with stable angina pectoris (AP) [129], in PBMCs of patients with CAD [130]. However, miR-24 levels reduced in blood of patients with atherosclerosis [131] and in plasma of patients with familial hypercholesterolemia (FH) [132].	MiR-24 directly suppressed the expression of SR-BI by binding to the 3′-UTR of mRNA, thus reducing the selective uptake of HDL-CE by HepG2 and THP-1 cells [128,133]. In addition, steroidogenesis reduced in steroidogenic cells [128].	In *ApoE^−/−^* mice, miR-24 reduced the expression of SR-BI and promoted the formation of atherosclerotic plaques [133].
miR-26a/b	*ABCA1*	The level of miR-26a-1 increased in plasma of patients with AMI [134]. The level of miR-26b increased in plasma of patients with UA [99], while miR-26a/b increased in EMPs of patients with UA [116]. Moreover, the expression of miR-26b was significantly upregulated in atherosclerotic plaques in humans [101]. However, miR-26b decreased in blood cells of patients with peripheral arterial disease (PAD) [121].	In RAW 264.7, THP-1, HEK293T and HepG2 cells, miR-26 bound to the 3′-UTR of *ABCA1* and suppressed its expression [135].	
miR-27a/b	*ABCA1*	The level of miR-27a increased in PBMCs of patients with CAD [32] and in plasma of patients with UA [99]. The level of miR-27b significantly increased in sclerotic intima samples and in serum of patients with atherosclerosis obliterans [136], in plasma of patients with AAA [137], as well as in PBMCs of the patients with CAD, and expression levels of miR-27b were significantly correlated with the severity of stenosis [123]. The level of miR-27b elevated in the liver of C57BL/6J mice, as well as in *ApoE^−/−^* female mice on a high-fat “Western” diet [138]. However, the decreased levels of miR-27b were observed in blood cells of patients with PAD [121] and in plasma of patients with CAD [111], as well as in aneurysm tissues of patients with AAA [137]. A reduced level of miR-27b is associated with heart failure, atherosclerosis, and the severity of PAD symptoms [139].	MiR-27a/b directly targeted the 3′-UTR of *ABCA1*, significantly reducing its mRNA and protein levels in foam cells derived from THP-1 and RAW 264.7, as well as in HepG2 cells [140]. MiR-27a/b also reduced cholesterol efflux from THP-1 macrophages to apoA-I through the suppression of *ABCA1*. A similar effect of miR-27b on *ABCA1* mRNA and protein levels and cholesterol efflux existed for Huh7 cells [141].	Modulation of miR-27b expression in wild-type mice regulated *ABCA1* expression in the liver but does not affect lipid levels [141].
miR-28	*ABCA1* ^1^	The level of miR-28-5p increased in patients with UA [142,143].	miR-28-5p targeted the signal-regulated kinase 2 (ERK2) and inhibited its expression that led to increase of *ABCA1* expression in THP-1 derived macrophages and HepG2 cells [142,143].	
miR-30e	*ABCA1*	The expression of miR-30e was significantly upregulated in the serum exosome of patients with CAD [26], in atherosclerotic plaques in humans [101], in plasma of patients with UA [99], and in blood cells of patients with AMI [120]. Moreover, miR-30e is considered as a differential biomarker for AMI [144]. However, there is evidence that miR-30e expression reduced in PBMCs of patients with lower extremities arterial disease (LEAD) [145] and in the whole blood of CAD patients [146].	MiR-30e directly targeted 3′-UTR of *ABCA1* and suppressed its protein expression [26].	
miR-34a	*ABCA1/ABCG1*	All studies evidence the increase of miR-34a in atherosclerosis- associated diseases. Thus, the level of miR-34a significantly increased in atherosclerotic plaques in humans and in *ApoE^−/−^* mice [147,148], in PBMCs of patients with LEAD [145], in plasma of patients with CAD [126,149] and AP [129]. Upregulated miR-34a is considered as a universal marker for AMI and UA [144].	In HepG2 cells, miR-34a directly interacted with the 3′-UTR of *ABCA1* and *ABCG1* mRNA and suppressed their expression [147]. Moreover, miR-34a inhibited cholesterol efflux from THP-1 and MPMs cells.	In mice, the downregulation of *ABCA1* and *ABCG1* by miR-34a promoted RCT suppression to plasma, liver and feces [147]. In *ApoE^−/−^* and *Ldlr^−/−^* mice, miR-34a promoted dyslipidemia, plaque growth, and instability.
miR-92a	*ABCA1*	The data on miR-92a expression in atherosclerosis are contradictory. The increased level of miR-92a was found in plasma and plasma exosomes of patients with the initial stage of atherosclerosis [150], with CAD [26,151], in aneurysm tissues of AAA [119], in human coronary atherosclerotic plaques [114], in plasma of patients with hypertension, especially with thickening of the carotid artery wall [152], in plasma of patients with UA [105], and with asymptomatic carotid artery stenosis, where it was correlated with the degree of stenosis [153], in PBMCs of CAD patients and in EMPs of patients with UA [116]. Moreover, upregulated miR-92a is considered as a differential biomarker for UA [144]. However, miR-92a expression decreased in the blood of patients with CAD [108,111,154], CHD [112] and atherosclerosis [155], in plasma and atherosclerotic plaques in PAD patients with cardiovascular events (CVEs) [156].	miR-92a directly targeted 3′-UTR of *ABCA1* and suppressed its protein expression [26].	Increased expression of miR-92a contributed to the development of atherosclerotic plaques under the influence of oxLDL in *Ldlr^−/−^* mice [157].
miR-93	*ABCA1*	Mostly, miR-93 levels increased in atherosclerosis. Thus, increased miR-93-5p level was detected in plasma of patients with critical coronary stenosis [158], with UA [105], CAD [159] and in blood cells of patients with AMI [120]. Moreover, miR-93 is considered as a universal biomarker for both AMI and UA [144]. However, miR-93 level decreased in CAD patients [160].	miR-93 directly targeted 3′-UTR of *ABCA1* and suppressed its protein expression [160].	
miR-96	*SCARB1*	MiR-96 level decreased in *ApoE^−/−^* mice on a high-fat diet [161]. The level of miR-96 was significantly upregulated in THP-1 cells stimulated to differentiate into macrophages.	miR-96 directly targeted 3′-UTR of *SCARB1*, suppressed its protein expression and HDL-C uptake by HepG2 and other human liver cells [161]. However, miR-96 increased HDL-C uptake by THP-1 cells, probably through the regulation of other pathways of cholesterol delivery.	
miR-101	*ABCA1*	IL-6 and TNF-α induced miR-101 expression in HepG2 cells and THP-1 macrophages [162]. During inflammation, miR-101 may promote the intracellular accumulation of lipids, which results in atherosclerosis.	MiR-101 directly interacted with the 3′-UTR of *ABCA1* and suppressed its protein expression, that reduced cholesterol efflux from cells to apoA-I [162].	
miR-106b	*ABCA1*	Level of miR-106b significantly decreased in plasma of patients with CAD and was correlated with HDL level [108]. MiR-106b level increased in plasma microparticles (MPs) of UA patients [105].	MiR-106b directly bound to the 3′-UTR of *ABCA1* and repressed its translation [163]. In neuronal cells (Neuro2a), miR-106b reduced ABCA1 levels and cholesterol efflux.	
miR-125a	*SCARB1*	miR-125a level decreased in the coronary arteries of patients with atherosclerotic plaques [164] and in the serum of patients with atherosclerosis [165] but increased in atherosclerotic plaques [101].	MiR-125a directly targeted 3′-UTR of *SCARB1* and suppressed SR-BI expression [166]. In rat/mouse Leydig tumor cells, suppression of SR-BI expression at mRNA and protein levels under the influence of miR-125a led to a decrease of HDL-CE uptake by cells and a decrease in HDL-dependent progesterone production. In mouse Hepa1-6 cells, miR-125a also suppressed SR-BI expression and HDL-CE uptake. However, in HepG2 cells, such effect of miR-125a was not found [161].	
miR-128	*ABCA1/ABCG1*	In mice on a high-fat diet, the level of miR-128 decreased in the liver, brain, and kidneys [167] but increased in the blood, brain, and heart [168]. miR-128-2 may prevent cholesterol efflux from cells at low cholesterol [167].	MiR-128-2 targeted 3′-UTR of *ABCA1* and *ABCG1* and inhibited their expression that led to the suppression of cholesterol efflux from HepG2, MCF7, and HEK293T cells [167]. Similar effects for miR-128-1 were found in mouse macrophages [169].	miR-128 is inversely correlated with *ABCA1* and *ABCG1* expression levels in different tissues of mice on a high-fat diet [167].
miR-130b	*ABCA1*		MiR-130b directly interacted with the 3′-UTR of *ABCA1* and suppressed its expression in HepG2 and in mouse macrophages, that led to reducing the cholesterol efflux [169].	
miR-143	*ABCA1*	MiR-143 was up-regulated and *ABCA1* was down-regulated in PAH patients [170]. MiR-143 level increased in human coronary atherosclerotic plaques [114].	MiR-143 directly suppressed the expression of *ABCA1* in pulmonary artery smooth muscle cells (PASMCs) [170].	MiR-143 promoted the development of hypoxia-induced pulmonary arterial hypertension (PAH) in vivo, presumably due to its influence on *ABCA1* expression [170]. The studies with *Ldlr^−/−^* and *Ldlr^−/−^* miR-143/145*^−/−^* double knockout mice revealed the contribution of these miRNAs to the development of atherosclerosis [171].
miR-144	*ABCA1*	MiR-144 increased in the plasma of patients with UA [99] and CAD [149,172,173], in monocytes of patients with hypertension [174]. However, miR-144 level was decreased in AAA tissue [137]. The level of miR-144 was associated with AMI [175]. LXR ligands increased the expression of miR-144 in mouse and human liver cells and macrophages, that may be important in homeostasis [176]. FXR transactivated miR-144 which suppressed *ABCA1* and cholesterol efflux [177].	MiR-144 directly interacted with the 3′-UTR of *ABCA1* and decreased its expression and cholesterol efflux to apoA-I [175,176,178].	miR-144 reduced the levels of *ABCA1* and HDL in the liver and plasma of mice [176,177]. In *ApoE^−/−^* mice, miR-144-3p decreased plasma HDL levels, impaired RCT and promoted the development of atherosclerosis [175]. A high-fat diet induced the development of atherosclerosis in miR-144*^−/−^* mice [179]. miR-144 promoted lipid accumulation and lipid disorder in F1-zebrafish [180].
miR-145	*ABCA1*	Data are contradictory. The level of miR-145 increased in the blood of patients with PAH [170], in plasma of patients with AMI [106] and within 24 h of stroke onset [127]. Upregulated level of miR-145 is considered as a biomarker for both AMI and UA [144]. The miR-145 levels are correlated with the size of the infarction area and may predict a long-term clinical outcome after AMI [181]. However, level of miR-145 decreased in the plasma of patients with AMI [182] and in the plasma and blood of patients with CAD, including very early onset [183], where it is correlated with disease severity [111,146,184].	MiR-145 targeted 3′-UTR of *ABCA1* and suppressed its protein expression and cholesterol efflux from HepG2 cells [178].	MiR-145 promoted a decrease in the ABCA1 protein in the mouse pancreas, as well as an increase in total cholesterol levels and a decrease in insulin secretion [178]. The studies in *Ldlr^−/−^* and *Ldlr^−/−^* miR-143/145*^−/−^* double knockout mice showed the contribution of these miRNAs to the development of atherosclerosis [171].
miR-148	*ABCA1*	The expression of miR-148b reduced in the serum of patients with atherosclerosis and in human aortic smooth muscle cells stimulated by ox-LDL [185]. The level of miR-148-3p increased in the liver of rhesus monkeys on a high-fat diet, as well as in mice (ob/ob) with genetically determined obesity [186].	MiR-148 directly bound the 3′-UTR of *ABCA1* and suppressed its expression [169,178,186]. As a result, miR-148 suppressed cholesterol efflux from HepG2 and mouse macrophages [169].	In C57BL/6J and *ApoE^−/−^* mice on a high-fat diet, miR-148 reduced liver ABCA1 and blood HDL [169]. In *Ldlr^−/−^* mice on a high-fat diet, miR-148 contributed to a decrease of ABCA1 in the liver and HDL in blood [186].
miR-183	*ABCA1*	In macrophages derived from THP-1, IL-18 promoted an increase in miR-183 expression with a concomitant decrease in *ABCA1* expression and cholesterol efflux, which may contribute to the development of atherosclerosis [187].	MiR-183 directly interacted with the 3′-UTR of *ABCA1* and suppressed its expression [187].	
miR-185	*SCARB1*	MiR-185-3p was upregulated in atherosclerotic mouse aorta [188]. miR-185 also increased in atherosclerotic plaques in humans [101]. However, in the liver of *ApoE^−/−^* mice on a high-fat diet, the miR-185 level decreased [161].	MiR-185 directly interacted with the 3′-UTR of *SCARB1* and suppressed the expression of SR-BI and HDL-C uptake in THP-1 cells and human hepatic cell lines [161].	
miR-188	*ABCA1*	MiR-188-3p decreased in *ApoE^−/−^* mice with atherosclerosis [189].		In *ApoE^−/−^* mice with atherosclerosis, miR-188-3p upregulated ABCA1 level in serum and promoted a decrease of lipid accumulation within the vessels and atherosclerosis [189].
miR-212	*ABCA1* ^1^	The miR-212 level decreased in plaques and macrophages of *ApoE^−/−^* mice on a high-fat diet [190].	In THP-1 macrophages, miR-212 targeted *SIRT1*, which led to inhibition of *ABCA1* expression, decreased cholesterol efflux and increased intracellular lipid accumulation [190].	
miR-223	*SCARB1/ABCA1* ^1^	miR-223 increased in CVD i.e., in *ApoE^−/−^* mice [191], in serum, in the vascular wall of patients with atherosclerosis obliterans [192], in the plasma of patients with AMI [115], PAD with cardiovascular events (CVEs) [156], unstable coronary artery disease (UCAD) [193], coronary artery calcification (CAC) [194] and UA [99,105], in platelets of patients with CAD [195], in atherosclerotic plaques of patients with PAD with cardiovascular events (CVEs) [156], and in aneurysm tissues of patients with AAA [119]. HDL-transported miR-223 elevated in patients with hypercholesterolemia and in *Ldlr^−/−^* and *ApoE^−/−^* mice on a high-fat diet. miR-223 increased in human hepatocytes with a high level of extracellular cholesterol [196]. An increased miR-223 level is associated with an increased risk of CVD [196]. MiR-223 expression is associated with atherogenesis in CAD [197]. However, the expression of miR-223 decreased in PBMCs of patients with CAD with the lowest stenosis less than 50% [198]. A reduced level of miR-223 is associated with heart failure, atherosclerosis, and the severity of PAD symptoms [139]. In THP-1 macrophages, miR-223 expression was significantly upregulated bur had no effect on *SCARB1* and HDL-C uptake [161]. A reduced cholesterol level caused a decrease in the level of miR-223 in J774 macrophages and Huh7 cells [199].	MiR-223 directly targeted the 3′-UTR of *SCARB1*, suppressed SR-B1 expression and the uptake of HDL-C in human hepatic cells [161,199]. miR-223 targeted Sp3, the repressor of Sp1-directed *ABCA1* transcription. Thus, miR-223 promoted the indirect increase of mRNA and protein levels of ABCA1, as well as the cholesterol efflux to apoA-I in Huh7 cells [199].	In *miR-223^−/−^* mice the level of SR-BI in the liver reduced, but total cholesterol and HDL-C increased in plasma. Cholesterol level increased in the liver of these mice [199].
miR-301b	*ABCA1*		MiR-301b directly bound to the 3′-UTR of *ABCA1* and suppressed its expression in HepG2 and mouse macrophages, that led to a decrease of cholesterol efflux [169].	
miR-302a	*ABCA1*	Ox-LDL downregulated miR-302a expression in mouse macrophages [200]. In the liver of *Ldlr^−/−^* mice on Western-type diet, miR-302a decreased [201].	MiR-302a targeted 3′-UTR of *ABCA1* and suppressed its protein expression in primary mouse and human macrophages, leading to suppression of cholesterol efflux [200].	In *Ldlr^−/−^* mice on an atherogenic diet, miR-302a suppressed *ABCA1* expression in the liver and aorta with a decrease of plasma HDL level, that promoted the growth of plaques, their instability and inflammation [200].
miR-361-5p	*ABCA1*		MiR-361-5p directly bound to the 3′-UTR of *ABCA1* and suppressed its expression [202].	
miR-378	*ABCG1*	MiR-378 levels increased in aortas during the progression of atherosclerosis in *ApoE^−/−^*mice [203]. Plasma miR-378 expression was significantly downregulated in patients with CAD [146,204], CHD [112]. Moreover, it is considered as biomarker for risk and severity of CHD [112].	MiR-378 directly interacted with the 3′-UTR of *ABCG1* and suppressed its expression that led to downregulation of cholesterol efflux from mouse and human macrophages [203].	In *ApoE^−/−^* mice, miR-378 presumably downregulated *ABCG1* expression in peritoneal macrophages, leading to decreased RCT and atherosclerosis progression [203].
miR-486	*ABCA1* ^1^	The level of miR-486 increased in the plasma of obese children and is associated with body mass index and other indicators of obesity [205]. The level of miR-486 elevated in the blood of patients with CAD [151] and is associated with the risk of developing cardiovascular diseases [109,206].	MiR-486 directly bound to 3′-UTR of histone acetyltransferase-1 (HAT1) and suppressed its expression with a concomitant decrease in ABCA1 expression at both mRNA and protein level, that led to cholesterol accumulation in THP-1 cells [207].	
miR-613	*ABCA1*	PPAR-γ, which induces the expression of a cascade of genes involved in cholesterol efflux from macrophages, negatively regulated the expression of miR-613 at transcriptional level [208].	miR-613 targeted 3′-UTR of *ABCA1* and suppressed its protein expression, which led to inhibition of cholesterol efflux from THP-1 cells activated by PPAR-γ [208].	
miR-758	*ABCA1*	The level of miR-758 decreased in cholesterol-enriched macrophages, as well as in pancreatic macrophages and liver cells in mice on a high-fat diet [209]. The level of miR-758 increased in plaques from patients with hypercholesterolemia compared to plaques of patients with normal cholesterol [210].	MiR-758 directly interacted with 3′-UTR of *ABCA1*, suppressed its expression and cholesterol efflux to apoA-I in mouse and human macrophages [209] and HepG2 cells [211].	

^1^ indirect target.
